# Plasma fatty acid composition predicts bone mineral accrual from childhood to adolescence: the Physical Activity and Nutrition in Children study

**DOI:** 10.1093/jbmr/zjaf104

**Published:** 2025-08-01

**Authors:** Timo A Lakka, Saara Heinonen, Taisa Sallinen, Aino-Maija Eloranta, Suvi Laamanen, Annie M Skinner, Eero A Haapala, Dimitris Vlachopoulos, Alan R Barker, Toni Rikkonen, Tomi P Laitinen, Jyrki Ågren, Sonja Soininen

**Affiliations:** Institute of Biomedicine, School of Medicine, Faculty of Health Sciences, University of Eastern Finland, 70211 Kuopio, Finland; Department of Clinical Physiology and Nuclear Medicine, Kuopio University Hospital, Wellbeing Services County of North Savo, 70210 Kuopio, Finland; Kuopio Research Institute of Exercise Medicine, 70210 Kuopio, Finland; Institute of Biomedicine, School of Medicine, Faculty of Health Sciences, University of Eastern Finland, 70211 Kuopio, Finland; Institute of Biomedicine, School of Medicine, Faculty of Health Sciences, University of Eastern Finland, 70211 Kuopio, Finland; Institute of Public Health and Clinical Nutrition, School of Medicine, Faculty of Health Sciences, University of Eastern Finland, 70210 Kuopio, Finland; Institute of Biomedicine, School of Medicine, Faculty of Health Sciences, University of Eastern Finland, 70211 Kuopio, Finland; Institute of Public Health and Clinical Nutrition, School of Medicine, Faculty of Health Sciences, University of Eastern Finland, 70210 Kuopio, Finland; Department of Medicine, Endocrinology and Clinical Nutrition, Kuopio University Hospital, Wellbeing Services County of North Savo, 70210 Kuopio, Finland; Institute of Public Health and Clinical Nutrition, School of Medicine, Faculty of Health Sciences, University of Eastern Finland, 70210 Kuopio, Finland; Children’s Health and Exercise Research Centre, University of Exeter, Exeter EX1 2LU, United Kingdom; Institute of Biomedicine, School of Medicine, Faculty of Health Sciences, University of Eastern Finland, 70211 Kuopio, Finland; Faculty of Sport and Health Sciences, University of Jyväskylä, 40014 Jyväskylä, Finland; Children’s Health and Exercise Research Centre, University of Exeter, Exeter EX1 2LU, United Kingdom; Children’s Health and Exercise Research Centre, University of Exeter, Exeter EX1 2LU, United Kingdom; Kuopio Musculoskeletal Research Unit (KMRU), University of Eastern Finland, 70210 Kuopio, Finland; Department of Clinical Physiology and Nuclear Medicine, Kuopio University Hospital, Wellbeing Services County of North Savo, 70210 Kuopio, Finland; Institute of Biomedicine, School of Medicine, Faculty of Health Sciences, University of Eastern Finland, 70211 Kuopio, Finland; Institute of Biomedicine, School of Medicine, Faculty of Health Sciences, University of Eastern Finland, 70211 Kuopio, Finland; Institute of Public Health and Clinical Nutrition, School of Medicine, Faculty of Health Sciences, University of Eastern Finland, 70210 Kuopio, Finland; Teaching Clinic Osmo, Wellbeing Services County of North Savo, 70210 Kuopio, Finland

**Keywords:** fatty acid, phospholipid, bone mineral density, dual-energy X-ray absorptiometry, child, adolescent

## Abstract

Little is known about the associations of plasma fatty acids (FAs) with bone mineral accrual, and the evidence is mostly based on cross-sectional data. In this observational study, we investigated for the first time the longitudinal associations of plasma FA composition as well as desaturase and elongase enzyme activities with BMD from childhood to adolescence. Altogether, 480 children (227 girls) aged 7-9 yr attending baseline examinations were included in the current analyses. The longitudinal associations of the proportions of FAs in plasma phospholipids, analyzed by gas chromatography, as well as estimated desaturase and elongase activities with total body less head BMD, measured by dual-energy X-ray absorptiometry, were analyzed by linear mixed-effects models using values from baseline, 2-yr, and 8-yr follow-up and adjusted for sex, maturity offset, follow-up time, and lean mass (LM) or fat mass (FM). Decreased proportion of linoleic acid (standardized regression coefficient *β* = −.023, *p* = .001), increased proportion of dihomo-gamma-linolenic acid (*β* = .029, *p* < .001), and Δ6-desaturase activity (*β* = .032, *p* < .001) were associated with increased BMD independent of sex, maturity offset, follow-up time, LM, and FM. Increased proportions of nervonic acid (*β* = .018, *p* = .012), arachidonic acid (*β* = .019, *p* = .017), and docosapentaenoic acid (*β* = .020, *p* = .013) were associated with increased BMD, and these associations were partly explained by LM. Increased proportions of arachidic acid (*β* = .022, *p* = .005), behenic acid (*β* = .018, *p* = .010), lignoceric acid (*β* = .015, *p* = .040), and palmitoleic acid (*β* = .016, *p* = .013), increased stearoyl-CoA-desaturase activity (*β* = .017, *p* = .009), and decreased elongase activity (*β* = −.017, *p* = .023) were associated with increased BMD, and these associations were partly explained by FM. Single plasma saturated, monounsaturated, and polyunsaturated FAs have divergent longitudinal associations with BMD from childhood to adolescence. Plasma FA composition predicts bone mineral accrual from childhood to adolescence, implying that FA metabolism is important for healthy bone development since childhood.

## Graphical Abstract

**Graphical Abstract:**
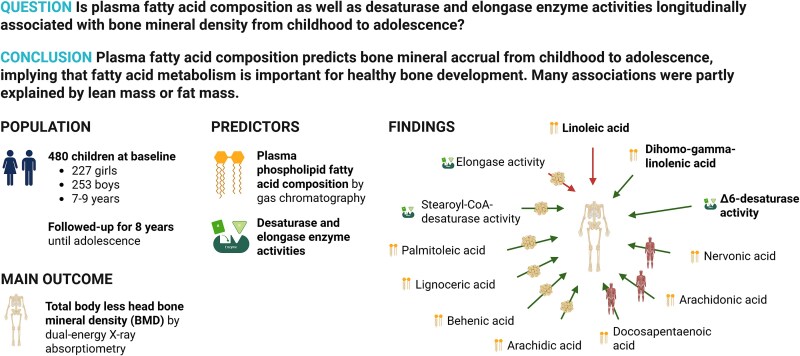

## Introduction

Childhood and puberty are the time periods of most rapid growth and bone accretion, and most bone mass is gained during adolescence and early adulthood.^1^ Low BMD tends to track from childhood into adulthood, and low peak bone mass in early adulthood is considered the strongest risk factor for developing osteoporosis.^1^ Therefore, it is important to recognize factors that influence bone mass accrual during childhood and adolescence beyond traditional factors such as dietary vitamin D and calcium intake, physical activity, and body composition.

Fatty acids (FAs) and related metabolites may play an important role in the development of osteopenia and osteoporosis, as they have both promotional and inhibitory actions on osteoblasts and osteoclasts by modulating bone metabolism via several mechanisms, including inflammation, oxidative stress, apoptosis, and autophagy.^2^ Some follow-up studies have shown that dietary FA intake influences BMD^3^ and predicts fracture risk in adults,^4^^,^^5^ but this evidence is inconsistent.^4–7^ Importantly, plasma FAs provide more objective information on FA availability for bone because they are less prone to measurement errors compared to dietary FA intake and are determined not only by diet but also by FA biosynthesis.^8^^,^^9^ However, few follow-up studies have investigated the associations of plasma FAs with BMD or the risk of fractures in adults, and the results of these longitudinal studies have been conflicting.^8^^,^^10^

There are few and mainly cross-sectional studies with small numbers of participants on the associations of dietary or circulating FAs with BMC and BMD in children and adolescents, and the results of these studies have been inconsistent.^11–14^ In a cross-sectional study among 85 children aged 8 yr, the proportions of a polyunsaturated FA (PUFA) arachidonic acid and a monounsaturated FA (MUFA) nervonic acid in serum phospholipids (PLs) were positively associated with BMC and BMD, whereas the proportions of a PUFA linoleic acid, a saturated FA (SFA) stearic acid, and total SFAs had weak inverse relationships with BMD.^11^ In another cross-sectional study among 111 children aged 4 yr, the proportion of a n-3 PUFA docosahexaenoic acid (DHA) in serum PLs was positively associated with BMD adjusted for age, sex, body weight, and socioeconomic status, but the associations of other PUFAs, including linoleic acid and arachidonic acid, and MUFAs, with BMD were largely explained by these confounding factors.^12^ The concentrations of n-3 PUFAs, especially DHA, and a n-6 PUFA arachidonic acid in serum PLs correlated positively with total and spine BMD at age 22 yr and with a change in spine BMD from age 16 to 22 yr in 78 men.^13^ However, a DHA-rich fish oil supplementation or the consequently increased proportion of DHA in blood erythrocytes had no effect on BMC, BMD, or plasma osteocalcin, a hormone produced by bones, in a 16-wk trial among 78 boys aged 13-15 yr.^14^

It remains unknown if plasma FAs predict the development of BMD from childhood to adolescence, which are critical periods for bone accretion and gaining bone mass. Therefore, we investigated the longitudinal associations of the proportions of FAs in plasma PLs as well as estimated desaturase and elongase enzyme activities with BMD in a general population of children followed up for 8 yr until adolescence. We also studied if potential confounding factors such as lean mass (LM), fat mass (FM), dietary calcium intake, or dietary vitamin D intake, explained these longitudinal associations.

## Materials and methods

### Study design and participants

The Physical Activity and Nutrition in Children (PANIC) study (ClinicalTrials.gov NCT01803776) is a non-randomized controlled trial to investigate the effects of a long-term physical activity and dietary intervention on cardiometabolic risk factors and other health outcomes in a general population of children from the city of Kuopio, Finland, followed up for 8 yr until adolescence. The Research Ethics Committee of the Hospital District of Northern Savo approved the study protocol in 2006 (Statement 69/2006) and in 2015 (422/2015). The parents gave written informed consent and their children provided assent to participation at baseline, and the adolescents and their parents gave written informed consent at the 8-yr follow-up. The PANIC study has been carried out in accordance with the principles of the Declaration of Helsinki as revised in 2008. All ethical regulations relevant to human research participants were followed.

**Figure 1:**
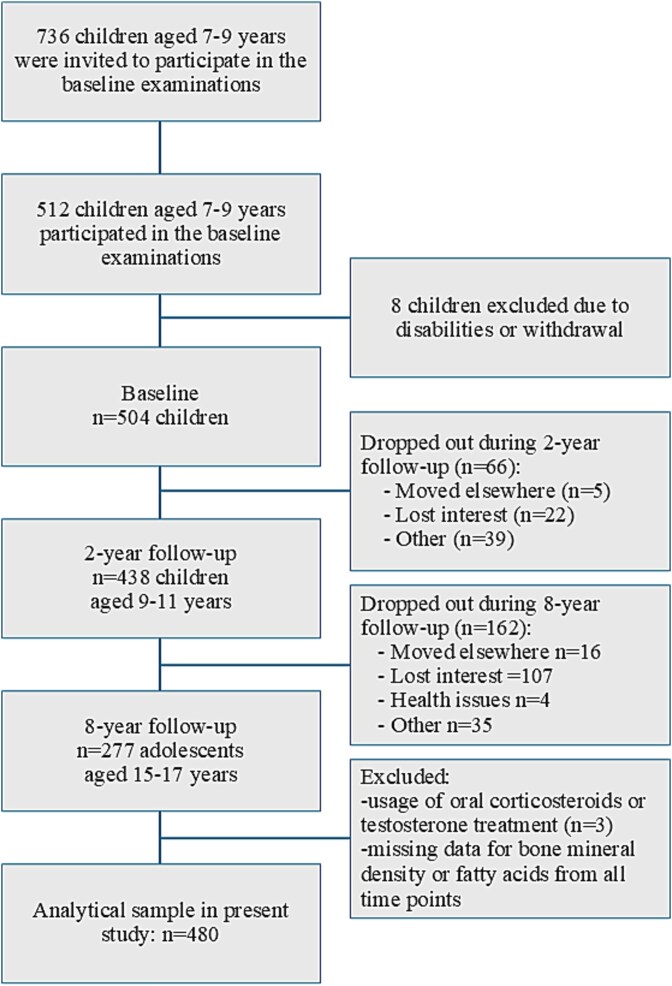
Flow chart.

We invited 736 children, 7-9 yr of age, who started the first grade in 16 primary schools of the city of Kuopio in 2007-2009. Altogether, 512 children (248 girls), representing 70% of those invited, participated in the baseline examinations in 2007-2009 (Figure 1). The participants did not differ in age, sex, or BMI-SD score (BMI-SDS) from all children who started the first grade in the city of Kuopio in 2007-2009 based on data from the standard school health examinations performed for all Finnish children. We excluded 6 children from the study at baseline owing to physical disabilities that could hamper participation in the intervention or no time or motivation to attend the study. We also excluded 2 children whose parents later withdrew their permission to use the data of their children. Of all 504 children who participated in the baseline examinations, 437 (87%) attended the 2-yr follow-up examinations and 277 (55%) attended the 8-yr follow-up examinations.

The intervention during the first 2 yr included 6 physical activity and dietary counseling sessions.^15^ After the 2-yr follow-up examinations, the physical activity and dietary intervention was continued less intensively with one annual counseling session until the 8-yr follow-up examinations.^16^ The participants and their parents in the control group received general verbal and written advice on health-improving physical activity and diet at baseline but no active intervention. The original sample size calculations of the PANIC study were based on the effect of a dietary intervention on fasting insulin, the main outcome of our intervention study, among children in a previous Finnish study.^17^

The current statistical analyses are based on the 8-yr follow-up design using data collected at the baseline, 2-yr, and 8-yr follow-up examinations of the PANIC study. Data on the proportions of FAs in plasma PLs and BMD were available for 490 and 465 participants at baseline, for 415 and 384 participants at the 2-yr follow-up, and for 263 and 263 participants at the 8-yr follow-up, respectively. We included in the statistical analysis 480 participants who had data on plasma FAs and BMD from at least 1 out of 3 time points and did not use oral corticosteroids or testosterone. Of these participants, 98.1% were Caucasian. Those who participated in the 8-yr follow-up examinations and were included in the current statistical analyses had similar age (mean 7.7 vs 7.6 yr, *p* = .258), height (mean 128.8 vs 128.8 cm, *p* = .923), weight (median 26.0 vs 25.8 kg, *p* = .504), BMI-SDS (mean − 0.12 vs -0.22, *p* = .316), and BMD (mean 0.72 vs 0.72 g/cm^2^, *p* = .723) but had slightly lower LM (mean 17.6 vs 18.0 kg, *p* = .047) and higher FM (median 4.7 vs 4.1 kg, *p* = .037) at baseline than those who did not attend the 8-yr follow-up examinations. The mean (SD) of follow-up time was 8.0 (0.2) years.

### Measurement of plasma FAs and enzyme activities

**Figure 2:**
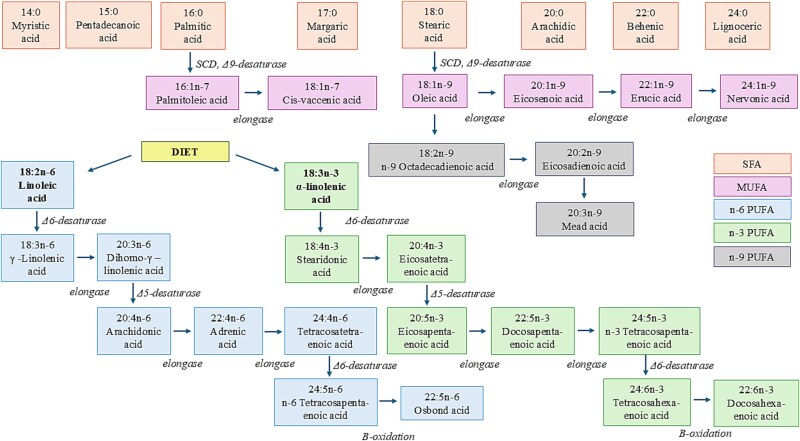
Desaturation and elongation of fatty acids.

Blood samples were collected after a 12-h overnight fast. Plasma FAs were analyzed by gas chromatography as described previously.^18^^,^^19^ Plasma samples were extracted with chloroform:methanol (2:1), and the lipid fractions were separated by solid-phase extraction with an aminopropyl column. FAs in plasma PLs were transmethylated with 14% boron trifluoride in methanol and were analyzed by the 7890A gas chromatograph (Agilent Technologies Inc., Wilmington, DE, USA) equipped with a 25-m FFAP column. Cholesteryl nonadecanoate (Nu Chek Prep Inc., Elysian, MA, USA), trinonadecanoin, and phosphatidylcholine dinonadecanoyl (Larodan Fine Chemicals, Malmö, Sweden) served as internal standards. The proportions of FAs in plasma PLs were expressed as mole percents of total PL FAs.^20^ Desaturase and elongase enzyme activities were estimated as the single product FA divided by its single precursor FA (Figure 2). Stearoyl-CoA-desaturase (SCD, Δ9-desaturase) activity was computed as 16:1n-7/16:0, Δ6-desaturase (D6D) activity as 20:3n-6/18:2n-6, Δ5-desaturase (D5D) activity as 20:4n-6/20:3n-6,^21^ and elongase activity as 18:1n-7/16:1n-7.^22^ The proportion of dihomo-gamma-linolenic acid (20:3n-6) was used to compute D6D activity because the proportion of gamma-linolenic acid in PLs was too low to detect and 20:3n-6/8:2n-6 has been found to be a valid estimate for D6D activity.^21^ We also computed the sum of n-6 PUFAs, the sum of n-3 PUFAs, and the n-6/n-3 PUFA ratio.

### Assessment of BMD and body size and composition

Body height and weight were assessed after a 12-h fast using a wall-mounted stadiometer and the InBody 720 bioelectrical impedance device (Biospace, Seoul, South Korea), respectively. BMI was calculated as weight (kg) divided by height squared (m^2^). We computed age- and sex-standardized BMI-SDS using Finnish references.^23^ Lean mass, FM, and total body less head BMD were assessed using the Lunar Prodigy Advance DXA device (GE Medical Systems, Madison, WI, USA) and the Encore software, Version 10.51.006 (GE Company, Madison, WI, USA), according to the manufacturer’s instructions using standardized protocols. The same DXA device and software were used in all measurements.

### Other assessments

General health was assessed by questionnaires completed by the parents at baseline and 2-yr follow-up and by the participants with parental assistance at 8-yr follow-up. The questionnaires included physician-diagnosed chronic diseases, allergies, and detailed medication use. A research physician conducted a clinical examination and assessed pubertal status using Tanner staging: breast development (M 1-5) for girls and testicular volume (G 1-5) measured by an orchidometer for boys.^24^^,^^25^ Maturity offset, which is a measure of somatic maturity, was calculated as −7.709133 + (0.0042232 × age × height) for girls and − 7.999994 + (0.0036124 × age × height) for boys in which age is expressed in years and height in centimeters.^26^ We assessed dietary calcium and vitamin D intake using 4-d food records as described in detail previously.^27^^,^^28^

### Statistical analyses

Statistical analyses were performed using the IBM SPSS Statistics software, Version 27 (IBM Corp., Armonk, NY, USA), R (R Core team, 2023), and R Studio (Rstudio Team, 2023). The normality of variable distributions was verified visually and by the Kolmogorov-Smirnov test. The *t*-test for independent samples, the Mann-Whitney U test, and the Pearson χ2 test were used to examine differences in the participant characteristics between sexes. Associations with a *p*-value of <0.05 were considered statistically significant.

The longitudinal associations of the proportions of FAs in plasma PLs with BMD were analyzed by linear mixed-effects models using the values of each FA, BMD, and confounding factors from all 3 time points. The linear mixed-effects models, which assume that the data are missing at random, are especially suitable for analyzing longitudinal datasets containing correlated and unbalanced data. We compared a random intercept model to a more complex model with both random intercept and slope, modeling individual variation over follow-up time from baseline. Model comparison using ANOVA function in R supported the random slope model. Model assumptions were evaluated using Q-Q and residual plots.

First, we adjusted the data for sex and maturity offset (model 1, BMD_ij_ = β_0_ + u_0i_ + (β_1_ + u_1i_)* follow-up time_ij_ + β_2_*sex_i_ + β_3_*maturity offset_ij_ + β_4_*FA*_ij_ + ε_ij_*), where BMD_ij_ is the observation for participant i at time j (baseline, 2-yr follow-up, and 8-yr-follow-up); *β*_0_ is the intercept; *β*_1_, *β*_2_, *β*_3_, and *β*_4_ are the regression coefficients for follow-up time, sex, maturity offset, and each FA, respectively; *u*_0*i*_ and *u*_1*i*_ are the participant specific random effects; and *ε_ij_* is the error for the participant *i* at time *j*. In similar linear mixed-effects models, we adjusted the data additionally for LM (model 2) or FM (model 3), as they have strong, positive, and independent associations with BMD in our study population of children.^29^ We also analyzed if sex modified the associations of the proportions of FAs in plasma PLs with BMD by including a sex*FA interaction term in the models. If sex modified any association, the analyses for models 1-3 were performed in girls and boys separately.

A systematic literature review concluded that potential benefits of n-3 FAs on skeletal health may be enhanced by concurrent administration of calcium.^7^ We have found that milk products, fat products, and fish are the main dietary sources of vitamin D^27^^,^^28^ and important dietary sources of FAs.^30^ Dietary fat also facilitates the absorption of vitamin D and other fat-soluble vitamins.^31^ As potential confounding factors, we therefore adjusted the data for dietary calcium intake or vitamin D intake in linear mixed-effects models among 441 participants with available dietary data (Table S1).

The physical activity and dietary intervention did not affect BMD over 2 or 8 yr, and further adjustment for the study group had no effect on the associations of the proportions of FA in plasma PLs with BMD. Therefore, we did not include the study group in the final models.

## Results

### Characteristics of participants

Girls were shorter and had lower body weight and LM, higher FM and maturity offset, and lower dietary calcium intake at baseline, 2-yr follow-up, and 8-yr follow-up than boys. Girls also had lower BMD, more advanced puberty, and lower dietary vitamin D intake at 2-yr and 8-yr follow-up, and lower dietary vitamin D intake at baseline and 8-yr follow-up than boys (Table 1). The FAs in plasma PLs were highest for palmitic acid, linoleic acid, stearic acid, and oleic acid (Table 2).

**Table 1:** Characteristics of participants.

	**All** ^a^	**Girls** ^a^	**Boys** ^a^	***p*-value**
**Age, y**				
**Baseline**	7.6 (0.4)	7.6 (0.4)	7.7 (0.4)	.306
**2-yr follow-up**	9.8 (0.4)	9.7 (0.4)	9.8 (0.4)	.309
**8-yr follow-up**	15.8 (0.4)	15.8 (0.4)	15.9 (0.5)	.077
**Height, cm**				
**Baseline**	128.8 (5.6)	127.9 (5.6)	129.7 (5.5)	<.001
**2-yr follow-up**	140.4 (6.3)	139.7 (6.5)	141.1 (6.0)	.028
**8-yr follow-up**	171.7 (8.5)	165.8 (5.6)	176.5 (7.3)	<.001
**Weight, kg**				
**Baseline**	26.0 (5.8)	25.5 (5.7)	26.6 (5.7)	.049
**2-yr follow-up**	33.0 (9.5)	32.3 (8.5)	34.0 (10.0)	.042
**8-yr follow-up**	59.6 (12.5)	56.7 (10.6)	63.5 (14.4)	<.001
**BMI-SDS**				
**Baseline**	−0.16 (1.08)	−0.16 (1.07)	−0.17 (1.09)	.875
**2-yr follow-up**	−0.13 (1.06)	−0.14 (1.02)	−0.11 (1.10)	.779
**8-yr follow-up**	−0.04 (1.02)	0.09 (0.88)	−0.14 (1.12)	.064
**BMD, g/cm** ^**2**^				
**Baseline**	0.722 (0.049)	0.718 (0.049)	0.724 (0.048)	.172
**2-yr follow-up**	0.791 (0.058)	0.784 (0.058)	0.797 (0.057)	.025
**8-yr follow-up**	1.034 (0.091)	1.007 (0.065)	1.057 (0.103)	<.001
**Fat mass, kg**				
**Baseline**	4.37 (3.56)	4.93 (3.63)	3.64 (3.44)	<.001
**2-yr follow-up**	6.75 (6.85)	7.00 (6.38)	6.48 (7.06)	.010
**8-yr follow-up**	12.52 (10.03)	15.55 (5.95)	8.19 (7.93)	<.001
**Lean mass, kg**				
**Baseline**	17.84 (2.25)	16.86 (2.00)	18.71 (2.09)	<.001
**2-yr follow-up**	21.82 (2.97)	20.72 (2.79)	22.87 (2.75)	<.001
**8-yr follow-up**	41.83 (8.26)	35.20 (3.81)	47.59 (6.60)	<.001
**Maturity offset**				
**Baseline**	−4.02 (0.51)	−3.59 (0.33)	−4.41 (0.28)	<.001
**2-yr follow-up**	−2.51 (0.66)	−1.96 (0.45)	−3.01 (0.35)	<.001
**8-yr follow-up**	2.65 (0.79)	3.33 (0.49)	2.10 (0.53)	<.001
**Tanner stage**				
**Baseline**				.051
** Stage 1**	466 (97.5%)	217 (96.0%)	249 (98.8%)	
** Stage 2**	12 (2.5%)	9 (4.0%)	3 (1.2%)	
** Stages 3-5**	0	0	0	
**2-yr follow-up**				<.001
** Stage 1**	311 (77.0%)	134 (66.7%)	177 (87.2%)	
** Stage 2**	93 (23.0%)	67 (33.3%)	26 (12.8%)	
** Stages 3-5**	0	0	0	
**8-yr follow-up**				.002
** Stages 1-2**	0	0	0	
** Stage 3**	20 (8.5%)	4 (3.6%)	16 (12.7%)	
** Stage 4**	136 (57.6%)	58 (52.7%)	78 (61.9%)	
** Stage 5**	80 (33.9%)	48 (43.6%)	32 (25.4%)	
**Dietary calcium intake, mg/d**				
**Baseline**	1177 (457)	1087 (392)	1257 (497)	<.001
**2-yr follow-up**	1158 (540)	1113 (499)	1214 (556)	.001
**8-yr follow-up**	1005 (658)	809 (564)	1124 (717)	<.001
**Dietary vitamin D intake, μg/d**				
**Baseline**	5.7 (2.8)	5.5 (2.2)	6.0 (3.3)	<.001
**2-yr follow-up**	7.7 (5.0)	7.6 (4.7)	7.8 (5.2)	.086
**8-yr follow-up**	9.2 (6.9)	6.9 (6.4)	11.0 (6.8)	<.001

The values are means (SDs) for normally distributed variables (age, height, BMI-SDS, bone mineral density, lean mass, maturity offset), medians (interquartile ranges) for skewed variables (weight, fat mass, dietary calcium intakeand dietary vitamin D intake), and numbers (percentages) of participants for categorical variable (Tanner stage). Differences between girls and boys were tested with independent samples *t*-test for normally distributed variables, Mann–Whitney’s U test for skewed variables and Pearson’s χ2-test for categorical variables. The *p*-values are for differences between girls and boys.

Abbreviation: BMI-SDS, BMI-SD score.

a Numbers of children and adolescents (*n*) vary from 236 to 480 in different variables: at baseline: *n* = 480, 227 girls and 253 boys: age, height, weight, maturity offset, BMI-SDS, *n* = 478, 226 girls and 252 boys: Tanner stage, *n* = 475, 225 girls and 250 boys: bone mineral density, fat mass, lean mass, *n* = 406, 194 girls and 212 boys: dietary calcium and vitamin D intake; at 2-yr follow-up: *n* = 421, 202 girls and 219 boys: age, height, weight, maturity offset, BMI-SDS, *n* = 404, 201 girls and 203 boys: Tanner stage, *n* = 405, 198 girls and 207 boys: bone mineral density, fat mass, lean mass, *n* = 376 175 girls and 201 boys: dietary calcium and vitamin D intake; and at 8-yr follow-up: *n* = 271, 122 girls and 149 boys: age, *n* = 270, 121 girls and 149 boys: height, weight, maturity offset, BMI-SDS, *n* = 260, 121 girls and 139 boys: bone mineral density, fat mass, lean mass, *n* = 236, 110 girls and 126 boys: Tanner stage, *n* = 226, 112 girls and 114 boys: dietary calcium and vitamin D intake.

### Longitudinal associations of SFAs in plasma PLs with BMD over 8 yr

Increased proportions of arachidic acid, behenic acid, and lignoceric acid in plasma PLs were associated with increased BMD adjusted for sex, maturity offset, and follow-up time (Table 3). All these associations strengthened slightly after further adjustment for LM but weakened markedly after adjustment for FM. Other SFAs were not associated with BMD adjusted for sex, maturity offset, and follow-up time. Increased total SFAs and stearic acid were associated with increased BMD only after adjustment for LM, and increased margaric acid with increased BMD only after adjustment for FM.

**Table 2:** Fatty acid composition of plasma phospholipids and estimated desaturase and elongase activities at baseline, 2-yr follow-up, and 8-yr follow-up.

**Fatty acids (mol%)**	**Baseline** **(*n* = 459)**	**2-yr follow-up (*n* = 381)**	**8-yr follow-up (*n* = 262)**
**Total SFA**	45.43 (0.83)	45.55 (1.02)	44.74 (1.09)
**14:0, myristic acid**	0.45 (0.13)	0.47 (0.17)	0.44 (0.11)
**15:0, pentadecanoic acid**	0.19 (0.05)	0.19 (0.07)	0.22 (0.04)
**16:0, palmitic acid**	29.15 (1.02)	29.00 (1.08)	28.90 (1.06)
**17:0, margaric acid**	0.34 (0.05)	0.33 (0.05)	0.39 (0.07)
**18:0, stearic acid**	13.42 (0.82)	13.66 (0.87)	12.94 (0.90)
**20:0, arachidic acid**	0.43 (0.07)	0.43 (0.07)	0.38 (0.06)
**22:0, behenic acid**	0.79 (0.15)	0.80 (0.15)	0.85 (0.14)
**24:0, lignoceric acid**	0.65 (0.14)	0.66 (0.15)	0.61 (0.12)
**Total MUFA**	14.26 (1.31)	14.18 (1.36)	14.71 (1.14)
**16:1n-7, palmitoleic acid**	0.57 (0.17)	0.57 (0.18)	0.52 (0.14)
**18:1n-9, oleic acid**	10.52 (1.25)	10.49 (1.36)	10.80 (1.09)
**18:1n-7, cis-vaccenic acid**	1.38 (0.16)	1.36 (0.16)	1.60 (0.20)
**20:1n-9 + 11, eicosenoic acid**	0.29 (0.04)	0.28 (0.04)	0.29 (0.04)
**24:1n-9, nervonic acid**	1.51 (0.34)	1.47 (0.31)	1.50 (0.26)
**Total PUFA**	40.31 (1.57)	40.27 (1.67)	40.56 (1.45)
***Total n6-PUFA***	32.70 (1.78)	32.77 (1.88)	33.62 (1.54)
**18:2n-6, linoleic acid**	20.96 (2.06)	21.10 (2.16)	21.62 (2.09)
**20:3n-6, dihomo-gamma-linolenic acid**	2.83 (0.48)	2.87 (0.50)	2.93 (0.58)
**20:4n-6, arachidonic acid**	8.37 (1.20)	8.28 (1.32)	8.56 (1.16)
**22:4n-6, adrenic acid**	0.32 (0.06)	0.31 (0.06)	0.30 (0.07)
**22:5n-6, osbond acid;**	0.22 (0.05)	0.21 (0.05)	0.21 (0.07)
***Total n3-PUFA***	7.61 (1.45)	7.50 (1.45)	6.94 (1.21)
**18:3n-3, alpha-linolenic acid**	0.40 (0.11)	0.41 (0.12)	0.43 (0.14)
**20:5n-3, eicosapentaenoic acid**	1.29 (0.53)	1.31 (0.53)	1.10 (0.42)
**22:5n-3, docosapentaenoic acid**	1.28 (0.20)	1.26 (0.23)	1.21 (0.23)
**22:6n-3, docosahexaenoic acid**	4.64 (1.03)	4.52 (1.03)	4.19 (0.91)
***n6/n3 PUFA ratio***	4.47 (0.99)	4.56 (1.01)	5.00 (0.94)
**Desaturase and elongase activities**			
**Stearoyl-CoA-desaturase (16:1n-7/16:0)**	0.02 (0.01)	0.02 (0.01)	0.02 (0.01)
**Δ5-desaturase (20:4n–6/20:3n–6)**	3.05 (0.75)	2.98 (0.74)	3.03 (0.72)
**Δ6-desaturase (20:3n–6/18:2n–6)**	0.14 (0.03)	0.14 (0.03)	0.14 (0.04)
**Elongase (18:1n–7/16:1n-7)**	2.63 (0.73)	2.57 (0.76)	3.31 (0.95)

The values are means (SDs). Abbreviations: mol%, molar percentage; SFA, saturated fatty acid; MUFA, monounsaturated fatty acid; PUFA, polyunsaturated fatty acid.

**Table 3:** Longitudinal associations of fatty acids in plasma phospholipids with bone mineral density over 8 yr.

**Fatty acids (mol%)**	**Model 1**		**Model 2**		**Model 3**	
	***β***	***p*-value**	***β***	***p*-value**	***β***	***p*-value**
**Total SFA**	0.012	0.068	0.021	**0.001**	0.000	0.952
**14:0, Myristic acid**	0.004	0.509	0.010	0.064	0.001	0.903
**15:0, Pentadecanoic acid**	0.005	0.353	0.008	0.127	0.005	0.359
**16:0, Palmitic acid**	−0.001	0.887	−0.005	0.494	0.004	0.588
**17:0, Margaric acid**	0.013	0.084	0.005	0.483	0.018	**0.013**
**18:0, Stearic acid**	0.008	0.287	0.019	**0.005**	−0.011	0.137
**20:0, Arachidic acid**	0.022	**0.005**	0.028	**<0.001**	0.013	0.068
**22:0, Behenic acid**	0.018	**0.010**	0.022	**0.001**	0.010	0.144
**24:0, Lignoceric acid**	0.015	**0.040**	0.022	**0.001**	0.009	0.164
**Total MUFA**	−0.005	0.460	−0.010	0.122	−0.002	0.728
**16:1n-7, Palmitoleic acid**	0.016	**0.013**	0.019	**0.002**	0.007	0.274
**18:1n-9, Oleic acid**	−0.010	0.131	−0.011	0.060	−0.007	0.284
**18:1n-7, Cis-vaccenic acid**	−0.004	0.626	−0.022	**0.002**	0.003	0.651
**20:1n-9 + 11, Eicosenoic acid**	−0.011	0.108	−0.020	**0.002**	0.003	0.636
**24:1n-9, Nervonic acid**	0.018	**0.012**	0.013	0.052	0.017	**0.016**
**Total PUFA**	−0.003	0.610	−0.004	0.461	0.001	0.817
***Total n-6 PUFA***	−0.009	0.152	−0.012	**0.045**	−0.003	0.630
**18:2n-6, Linoleic acid**	−0.023	**0.001**	−0.021	**0.001**	−0.014	**0.031**
**20:3n-6, Dihomo-gamma-linolenic acid**	0.029	**<0.001**	0.023	**0.001**	0.014	**0.050** ^a^
**20:4n-6, Arachidonic acid**	0.019	**0.017**	0.012	0.124	0.019	**0.013**
**22:4n-6, Adrenic acid**	0.014	0.077	0.004	0.602	0.016	**0.032**
**22:5n-6, Osbond acid**	−0.008	0.350	−0.0068	0.392	−0.011	0.190
***Total n-3 PUFA***	0.010	0.174	0.012	0.061	0.006	0.339
**18:3n-3, Alpha-linolenic acid**	−0.006	0.376	−0.005	0.464	−0.005	0.480
**20:5n-3, Eicosapentaenoic acid, EPA**	0.006	0.362	0.009	0.117	0.001	0.859
**22:5n-3, Docosapentaenoic acid, DPA**	0.020	**0.013**	0.012	0.101	0.017	**0.030**
**22:6n-3, Docosahexaenoic acid, DHA**	0.008	0.203	0.011	0.113	0.007	0.345
***n-6/n-3 PUFA ratio***	−0.014	0.067	−0.017	**0.015**	−0.008	0.280
**Desaturase and elongase activities**						
**Stearoyl-CoA-desaturase (16:1n-7/16:0)**	0.017	**0.009**	0.020	**0.001**	0.007	0.265
**Δ5-desaturase (20:4n–6/20:3n–6)**	−0.009	0.203	−0.010	0.156	0.001	0.913
**Δ6-desaturase (20:3n–6/18:2n–6)**	0.032	**<0.001**	0.027	**<0.001**	0.018	**0.010**
**Elongase (18:1n–7/16:1n-7)**	−0.017	**0.023**	−0.030	**<0.001**	−0.003	0.653

The values are standardized regression coefficients from linear mixed-effects models adjusted for sex, maturity offset, and follow-up time (model 1); sex, maturity offset, follow-up time, and lean mass (model 2); or sex, maturity offset, follow-up time, and fat mass (model 3). *p*-values indicating statistically significant associations are bolded. Abbreviations: mol%, molar percentage; SFA, saturated fatty acid; MUFA, monounsaturated fatty acid; PUFA, polyunsaturated fatty acid.

a Exact *p*-value is .0498.

### Longitudinal associations of MUFAs in plasma PLs with BMD over 8 yr

Increased proportion of palmitoleic acid and nervonic acid in plasma PLs were associated with increased BMD after adjustment for sex, maturity offset, and follow-up time (Table 3). The association of palmitoleic acid strengthened slightly and the association of nervonic acid weakened markedly after further adjustment for LM. The association of palmitoleic acid weakened markedly after adjustment for FM. Decreased cis-vaccenic acid was associated with increased BMD only after further adjustment for LM, and decreased eicosenoic acid (gondoic acid) was associated with increased BMD after additional adjustment for LM (Table 3), dietary calcium intake, or dietary vitamin D intake (Table S1). Total MUFAs and oleic acid were not associated with BMD.

### Longitudinal associations of PUFAs in plasma PL with BMD over 8 yr

A decreased proportion of linoleic acid and increased proportions of dihomo-gamma-linolenic acid, arachidonic acid, and docosapentaenoic acid (DPA) in plasma PLs were associated with increased BMD after adjustment for sex, maturity offset, and follow-up time (Table 3). The associations of linoleic acid and dihomo-gamma-linolenic acid with BMD remained similar but the association of DPA with BMD weakened after further adjustment for LM. The associations of linoleic acid and dihomo-gamma-linolenic acid weakened markedly and the association of DPA weakened slightly but remained statistically significant after adjustment for FM. Other PUFAs were not associated with BMD adjusted for sex, maturity offset, and follow-up time. However, decreased total n-6 PUFAs and a decreased n-6/n-3 PUFA ratio were associated with increased BMD after additional adjustment for LM, and increased adrenic acid was associated with increased BMD after adjustment for FM.

### Longitudinal associations of desaturase and elongase activities with BMD over 8 yr

Increased SCD and D6D and decreased elongase activities were associated with increased BMD adjusted for sex, maturity offset, and follow-up time (Table 3). The associations of SCD and elongase activities with BMD strengthened after further adjustment for LM. The association of D6D activity with BMD weakened but remained statistically significant, whereas the association of SCD and elongase activities weakened markedly after adjustment for FM. The association of SCD activity with BMD strengthened slightly after adjustment for dietary calcium or vitamin D intake (Table S1). D5D activity was not associated with BMD (Table 3).

### Longitudinal associations of FAs in plasma PLs with BMD over 8 yr in girls and boys separately

The associations of all FAs as well as desaturase and elongase activities with BMD were similar in girls and boys after adjustment for sex, maturity offset and follow-up time, except that the proportion of margaric acid in plasma PLs was positively associated with BMD in boys but not in girls (boys: standardized regression coefficient *β* = .033, *p* = .003; girls: *β* = −.009, *p* = .355; *p* < .001 for interaction) and elongase activity was negatively associated with BMD in girls but not in boys (girls: *β* = −.029, *p* = .008; boys: *β* = −.013, *p* = .188; *p* = .007 for interaction). These associations were also similar in girls and boys after further adjustment for LM, except that osbond acid was negatively associated with BMD in girls but not in boys (girls: *β* = −.026, *p* = .013; boys: *β* = .003, *p* = .806; *p* = .041 for interaction). These associations were also similar in girls and boys after adjustment for FM, except that margaric acid was positively associated with BMD in boys but not in girls (boys: *β* = .0393, *p* < .001; girls: *β* = −.0028, *p* = .739; *p* < .001 for interaction).

## Discussion

We showed here for the first time that plasma FA composition predicts bone mineral accrual from childhood to adolescence, implying that FA metabolism is important for healthy bone development. We found that a decreased proportion of linoleic acid and an increased proportion of dihomo-gamma-linolenic acid in plasma PLs as well as increased D6D activity were associated with increased BMD independent of LM and FM over 8 yr from childhood to adolescence. Increased proportions of nervonic acid, arachidonic acid, and DPA in plasma PLs were associated with increased BMD over 8 yr, but these associations were partly explained by LM. Moreover, increased proportions of behenic acid, arachidic acid, lignoceric acid, and palmitoleic acid in plasma PLs, increased SCD activity, and decreased elongase activity were associated with increased BMD over 8 yr, but these associations were partly explained by FM.

Saturated FAs may impair bone and cartilage metabolism by increasing oxidative stress and inflammation.^2^ However, no association between total SFAs in plasma PLs and future fracture risk was reported in a longitudinal study among older adults,^8^ and no difference in plasma total SFAs was found between postmenopausal women with normal, osteopenic, and osteoporotic BMD in a cross-sectional study, either.^32^ We observed a positive association of total SFAs and stearic acid with BMD, but only controlling for LM. Interestingly, single FAs seem to be heterogenous in their biological effects, as differences in plasma palmitic and lignoceric acid but no other SFAs were observed between postmenopausal women with normal, osteopenic, and osteoporotic BMD.^32^ An inverse association of plasma stearic acid and a positive association of plasma arachidic acid with BMD was reported in a cross-sectional study among 8-yr-old children,^11^ but many SFAs such as margaric, behenic, and lignoceric acid were not measured. Behenic acid in erythrocyte PLs was inversely associated with calcaneus stiffness index, a measure of bone strength and a predictor for fracture risk, in Inuit women.^33^ However, plasma behenic acid was not associated with BMD in another cross-sectional study among postmenopausal women.^32^ We found no longitudinal studies on the associations of behenic acid, or even total SFAs, with BMD and no cross-sectional or longitudinal studies on the association of margaric acid (heptadecanoid acid) with BMD. We observed that increased PL arachidic, behenic, and lignoceric acids were longitudinally associated with increased BMD from childhood to adolescence. However, the associations of arachidic, behenic, and lignoceric acids were partly explained by FM. We have previously found that sphingomyelin-derived plasma arachidic, behenic, and lignoceric acids were inversely associated with plasma triglycerides in the current study population of children.^34^ This suggests that the relative amount of sphingomyelin in plasma PLs decreases with increasing plasma triglycerides or that increased FA and triglyceride synthesis in the liver affects the FA composition of sphingomyelins. Taken together, these observations suggest that the FA composition of sphingomyelin is associated with plasma triglyceride concentrations, potentially via hepatic lipid metabolism and VLDL production. Notably, the associations of sphingomyelin-derived FAs with BMD weakened after controlling for FM, indicating that adiposity may act as a confounding or mediating factor in these relationships.

Margaric acid, which was positively associated with BMD among boys in the current longitudinal study, is considered a marker of dairy fat intake.^9^ However, it can also be synthesized endogenously from gut-derived propionate, a marker of dietary fiber intake.^35^ We previously observed cross-sectional associations of higher consumptions of milk, low-fat sour milk products, fatty cheese, butter and butter-oil mixtures, and high-fiber grain products and lower consumptions of candy, chocolate, and hot chocolate powder with higher PL margaric acid in the current study population of children.^36^ The intakes of skimmed milk and sour milk products were also positively associated with PL behenic acid and arachidic acid.^36^ The positive longitudinal association of PL arachidic acid with BMD in our current study was independent of dietary calcium and vitamin D intake, both of which are known to help reach sufficient bone mass and BMD from childhood to adulthood.^1^ The results of these studies together suggest that higher consumptions of milk products and foods rich in fiber are beneficial and a higher consumption of candy is disadvantageous for bone mineral accrual. As the evidence on the longitudinal associations of various plasma SFAs with BMD remains limited and inconsistent, more follow-up studies in different age groups are warranted.

Long-chain MUFAs have been suggested to promote bone formation and inhibit bone degeneration.^2^ Gene variants related to higher levels of palmitoleic acid and oleic acid in plasma PLs were associated with higher estimated BMD, assessed by heel quantitative ultrasound, and a lower fracture risk in a Mendelian randomization study using adult data from the UK Biobank.^37^ However, the prevalence of osteopenia was increased in those with the highest plasma levels of MUFAs in a cross-sectional study among postmenopausal women,^32^ and increased PL MUFAs predicted an increased fracture risk in a longitudinal study among elderly men but not women.^8^ In our current longitudinal study, PL total MUFAs did not predict BMD from childhood to adolescence. Consistently with the results of a Mendelian randomization study,^37^ however, we found a positive, longitudinal association between the proportion of PL palmitoleic acid and BMD, although the association was partly explained by FM. In contrast, a small cross-sectional study among 4-yr-old children reported an inverse correlation between serum PL palmitoleic acid and lumbar BMD, but many potential confounding factors were not considered.^12^ Consistent with the result of a cross-sectional study in 8-yr-old children,^11^ we found a direct longitudinal association between a MUFA nervonic acid and BMD from childhood to adolescence. Previously, we have observed a direct cross-sectional association between skimmed milk consumption and plasma nervonic acid^36^ and that skimmed milk is a major source of calcium and vitamin D^30^ in the present pediatric study population. These findings could partly explain the positive longitudinal association between plasma nervonic acid and BMD. However, this association persisted after controlling for dietary calcium and vitamin D intake, which implies that not only diet but also FA metabolism may partly explain the observed longitudinal association between plasma nervonic acid and BMD from childhood to adolescence. Because of the limited and inconsistent evidence on the longitudinal associations of plasma MUFAs with BMD, more follow-up studies in different age groups and studies on potential mechanisms for these associations are needed.

Long-chain n-6 PUFAs, together with long-chain n-3 PUFAs, are precursors of eicosanoids, comprising prostaglandins, leukotrienes, and thromboxanes, which have hormone-like activities and may affect bone health in several complex ways.^38^ Long-chain n-6 PUFAs may increase pro-inflammatory cytokines, which increase bone resorption.^2^ Consistent with the results of cross-sectional studies in children 8 yr of age^11^ and in adults,^10^^,^^39^ we found an inverse longitudinal association between PL linoleic acid and BMD from childhood to adolescence. In a small study among 4-yr-old children, serum PL linoleic acid was inversely associated with BMD only among overweight children.^12^ We observed that the inverse association between linoleic acid and BMD attenuated slightly after controlling for FM. A circulating proportion of linoleic acid has been shown to reflect well the dietary intake of linoleic acid.^9^ We previously found that the intake of skimmed milk was inversely and the intake of vegetable-oil based margarine was positively associated with linoleic acid in plasma PLs.^36^ However, the inverse association of linoleic acid even strengthened slightly after controlling for dietary calcium and vitamin D intake, implying that the association is independent of these nutrients. In cross-sectional studies among adults, circulating gamma-linolenic acid, an intermediate in the n-6 pathway from linoleic acid to dihomo-gamma-linolenic acid, has been inversely associated with BMD,^39^ or no association with BMD has been found.^40^ We did not detect gamma-linolenic acid in plasma PLs but found that PL dihomo-gamma-linolenic acid was positively associated with BMD in our longitudinal study. Arachidonic acid, a product of dihomo-gamma-linolenic acid, was also positively associated with BMD in the current study, which is consistent with the results of a small longitudinal study among young men^13^ and cross-sectional studies among children and adolescents.^11^^,^^12^ In accordance with our findings, higher plasma phosphatidylcholine arachidonic acid was also associated with lower fracture risk in adults.^10^ We found no association between PL total n-6 PUFAs and BMD, which is reasonable as linoleic acid, the most abundant PL n-6 PUFA in our study population, had an inverse association whereas other n-6 PUFAs had positive associations with BMD.

It has been suggested that n-3 PUFAs can increase the absorption of calcium from the intestine and decrease urinary calcium excretion.^2^ Moreover, n-3 PUFAs may affect bone metabolism through their immune-modulating and anti-inflammatory actions, such as decreasing pro-inflammatory cytokines, leading to lower bone resorption.^2^^,^^7^ In cross-sectional studies, higher plasma n-3 PUFAs have been associated with higher spinal and femoral neck BMD among postmenopausal women,^32^ whereas serum n-3 PUFAs were not related to BMD among 8-yr old children.^11^ In our longitudinal study, PL total n-3 PUFAs was not associated with BMD. Of single n-3 PUFAs, DPA, a precursor for DHA and a product of EPA, was positively associated with BMD, consistent with the results of a large cross-sectional study in adults.^40^ DPA can be derived from fish, but interestingly, fish-oil supplementation only slightly increased the PL DPA, and more strongly increased PL EPA and DHA.^41^ Moreover, fish intake was not cross-sectionally associated with PL DPA among children at the baseline of our study.^36^ EPA and DHA supplementation trials have shown dose dependent increases in the proportions of these FAs in plasma PLs and cholesteryl esters, but also an increase in the proportion of DPA despite its low dose in supplements.^9^ These results suggest that an increase in plasma DPA could result from a higher conversion of EPA to DPA or a lower conversion of DPA to DHA. We found no longitudinal associations of other n-3 PUFAs with BMD from childhood to adolescence. Serum PL total n-3 PUFAs, especially DHA were positively associated with BMD at age 22 yr and with an increase in spine BMD from age 16 to 22 yr in men.^13^ However, no such association was found between DPA and BMD in these men. Serum DHA correlated positively with BMD also in 4-yr-old children,^12^ whereas fish oil supplementation and erythrocyte DHA were not associated with bone mass or the biomarkers of bone formation in adolescent boys.^14^ Plasma phosphatidylcholine DHA was also inversely associated with BMD in women but positively related to BMD in men.^10^ Taken together, our findings and the results of previous studies suggest that marine n-3 PUFAs EPA, DPA, and DHA may be beneficial for bone health. However, as some inconsistency exists in these findings, more follow-up studies in different age groups are needed.

Linoleic acid and alpha-linolenic acids are essential FAs that serve as precursors for long-chain n-6 and n-3 PUFAs, respectively, via elongation and desaturation reactions.^38^ Excessive dietary linoleic acid intake can raise its blood levels and lead to the formation of oxidized linoleic acid metabolites with adverse health effects.^42^ As discussed earlier, n-6 and n-3 PUFAs may have opposite effects on pro-inflammatory cytokines,^2^ making their balance important for a cytokine profile favorable to bone remodeling.^7^ Moreover, n-3-PUFAs have been shown to decrease the production of prostaglandin E2, derived from the n-6 PUFA arachidonic acid.^2^^,^^7^ Prostaglandin E2 may promote bone formation via activation of osteoblasts but also enhance bone resorption through dose-dependent effect on osteoclasts.^2^^,^^7^ In line with the results of a previous cross-sectional study in children aged 8 yr,^11^ we found an inverse longitudinal association between plasma n-6/n-3 PUFA ratio and BMD from childhood to adolescence, although it was evident only after controlling for LM. In conclusion, while adequate linoleic acid is essential, a higher proportion of linoleic acid—especially when combined with low n-3 PUFA levels—may be detrimental to bone health.

We found that D6D activity, estimated as the ratio of dihomo-gamma-linolenic acid and linoleic acid,^21^ was positively associated with BMD, independent of FM, LM, as well as dietary calcium and vitamin D intake. To our knowledge, no previous studies have examined the association between D6D activity and BMD. Desaturation and elongation are key lipid metabolism steps that modify dietary and endogenous SFAs. These steps convert SFAs into MUFAs and PUFAs from essential dietary FAs α-linolenic acid (n-3) and linoleic acid (n-6). These transformations primarily occur in the liver and adipose tissue. D6D is involved in the desaturation of n-6 and n-3 PUFAs. In the current study, the positive association between D6D activity and BMD appears to result from the negative association of linoleic acid and the positive association of dihomo-gamma-linolenic acid with BMD. This may also imply that the desaturation of FAs into longer-chain FAs is important for bone health. SCD has been suggested to be involved in bone homeostasis by promoting osteogenic differentiation of bone marrow mesenchymal stem cells^43^ and by affecting Wnt signaling.^44^ In a cross-sectional Mendelian randomization study in adults, higher plasma levels of a MUFA oleic acid, indicating higher SCD activity, were associated with higher BMD and a lower fracture risk.^37^ Moreover, a gene variant in PKD2L1 locus was suggested to impact heel bone stiffness index via modification of SCD activity, as its minor allele was associated with decreased SCD mRNA expression in visceral adipose tissue, and higher mRNA expression of SCD in visceral adipose tissue was associated with a higher heel stiffness index.^45^ In line with the results of that study, we observed a direct longitudinal association between SCD activity and BMD, although it attenuated after controlling for FM. On the other hand, SCD activity has been associated positively with fracture risk in a longitudinal study among men.^46^ SCD is a central regulator of lipid metabolism and fat storage, catalyzes the rate-limiting step in the biosynthesis of MUFAs from SFAs, has a role in lipogenesis, and is highly expressed in adipose tissue.^45^ Increased SCD may thereby reflect higher body mass due to adiposity, but on the other hand, inflammation that is related to higher adiposity may confound the association between SCD and BMD. We also found an inverse association between elongase activity and BMD, but this association attenuated after controlling for FM. This suggests that adiposity partly explains the association between elongase activity and BMD.

Major strengths of this study include the general population of children followed up for 8 yr until adolescence and the assessment of plasma FA composition by gas chromatography and BMD by whole body DXA using values from 3 time points over the long-term follow-up study. Childhood and adolescence are critical for bone formation, making it essential to identify factors affecting peak bone mass, the most important determinant of lifelong skeletal health. Another strength of our study is the smaller number of potential confounders, such as medications, smoking, and clinical conditions affecting bone health in youth compared to adults. Moreover, adults have already yielded their peak bone mass, and postmenopausal women and older adults are in the phase of decreasing bone mass. In addition to their effects on bone formation and bone health, sex and aging have been shown to affect plasma FA composition.^9^ However, the associations of plasma FAs with BMD were similar among girls and boys in the current study, with few sex differences observed. To consider several possible confounding factors, we controlled for sex, maturity offset, follow-up time, LM, FM, as well as dietary calcium and vitamin D intake in the statistical analyses. Measuring plasma FA composition with gas chromatography is a more objective method to assess FAs available to peripheral tissues than dietary FA intake as circulating FAs are determined not only by dietary fat and carbohydrate intake but also by FA biosynthesis.^9^^,^^36^ Moreover, dietary intakes may be under- or overestimated, and relative dietary intakes of single FAs are difficult to estimate.^9^ Another issue that should be considered when interpreting the results of different studies is that FA composition varies across lipid pools, including adipose tissue, plasma, serum, erythrocytes, and platelet lipids.^9^ We measured the FA composition of plasma PLs, which has been used in many large epidemiological studies, and the FA composition in the current study was similar to that in previous studies.^9^ Assessing and interpreting BMD in children is challenging due to individual variation in maturation and growth as well as for methodological reasons. We have measured BMD in children and adolescents using total body less head DXA, which is the most widely used and recommended method to evaluate BMD by the International Society of Densitometry^47^^,^^48^ and is also well reproducible in children and adolescents.^48^^,^^49^ Areal BMD may underestimate the BMD of short children and overestimate the BMD of tall children.^48^ Therefore, the International Society of Densitometry recommends adjusting BMD for height *z*-score in short children. As we investigated children and adolescents longitudinally using 3 time points, we adjusted the data not only for sex, but also maturity offset to consider growth and maturity.

Our study also has some limitations. Although we adjusted the data for several possible confounding factors, residual confounding remains a limitation of our study like in all observational studies, and causality of the associations cannot be assumed. Dietary intake data were not available for all participants, which slightly limits statistical power in the analyses controlling for dietary calcium and vitamin D intake. Although linear mixed-effects models allow for missing data and make use of all available observations, the smaller sample size at the 8-yr follow-up may reduce statistical power and increase estimate uncertainty at that time point. However, the differences in the baseline characteristics between the participants who attended the 8-yr follow-up and those who did not were minor. Therefore, we believe that the missing data do not markedly affect result interpretation. Finally, given the number of associations tested, some statistically significant associations found may have occurred due to chance.

## Conclusions

To the best of our knowledge, this is the first longitudinal study in a general population to show that FA composition in plasma PLs predicts bone mineral accrual from childhood to adolescence, implying that FA metabolism is important for healthy bone development. The proportion of linoleic acid in plasma PLs was negatively and the proportion of dihomo-gamma-linolenic acid in plasma PLs and D6D activity were positively associated with BMD independent of LM, FM, as well as dietary calcium and vitamin D intake. The positive associations of nervonic acid, arachidonic acid, and DPA with BMD were partly explained by LM. The positive associations of arachidic acid, behenic acid, lignoceric acid, palmitoleic acid, and SCD activity with BMD as well as the negative association of elongase activity with BMD were partly explained by FM. Dietary calcium and vitamin D intake did not markedly affect the associations of these FAs with BMD. Single SFAs, MUFAs, and PUFAs showed divergent associations with BMD, implying that the biological effects of FAs within these structural groups may not be metabolically equivalent. Therefore, more long-term follow-up studies on the longitudinal associations of single circulating FAs with bone health and on dietary factors affecting plasma FA composition in different age groups are warranted.

## Supplementary Material

Lakka_et_al_Supplemental_Table_revised_clean_zjaf104

## Data Availability

The data underlying this article cannot be shared publicly as it contain information that could compromise research participant privacy or consent. PANIC is an ongoing study, and therefore, the data are not fully anonymized and not openly available. The data will be shared on reasonable request to the principal investigator of the PANIC study [T.A.L.].

## References

[1] Weaver CM, Gordon CM, Janz KF, et al. The National Osteoporosis Foundation’s position statement on peak bone mass development and lifestyle factors: a systematic review and implementation recommendations. Osteoporos Int. 2016;27(4):1281–1386. 10.1007/s00198-015-3440-3 26856587 PMC4791473

[2] Bao M, Zhang K, Wei Y, et al. Therapeutic potentials and modulatory mechanisms of fatty acids in bone. Cell Prolif. 2020;53(2):e12735. 10.1111/CPR.12735 PMC7046483 31797479

[3] Järvinen R, Tuppurainen M, Erkkilä AT, et al. Associations of dietary polyunsaturated fatty acids with bone mineral density in elderly women. Eur J Clin Nutr. 2012;66(4):496–503. 10.1038/EJCN.2011.188 22113249

[4] Benetou V, Orfanos P, Zylis D, et al. Diet and hip fractures among elderly Europeans in the EPIC cohort. Eur J Clin Nutr. 2011;65(1):132–139. 10.1038/EJCN.2010.226 20948558

[5] Orchard TS, Cauley JA, Frank GC, et al. Fatty acid consumption and risk of fracture in the Women’s health initiative. Am J Clin Nutr. 2010;92(6):1452–1460. 10.3945/AJCN.2010.29955 20980487 PMC2980969

[6] Virtanen JK, Mozaffarian D, Willett WC, Feskanich D. Dietary intake of polyunsaturated fatty acids and risk of hip fracture in men and women. Osteoporos Int. 2012;23(11):2615–2624. 10.1007/S00198-012-1903-3/TABLES/3 22270860 PMC4213390

[7] Orchard TS, Pan X, Cheek F, Ing SW, Jackson RD. A systematic review of omega-3 fatty acids and osteoporosis. Br J Nutr. 2012;107(0 2):S253. 10.1017/S0007114512001638 22591899 PMC3899785

[8] Harris TB, Song X, Reinders I, et al. Plasma phospholipid fatty acids and fish-oil consumption in relation to osteoporotic fracture risk in older adults: the age, gene/environment susceptibility study. Am J Clin Nutr. 2015;101(5):947–955. 10.3945/AJCN.114.087502 25787995 PMC4409686

[9] Hodson L, Skeaff CM, Fielding BA. Fatty acid composition of adipose tissue and blood in humans and its use as a biomarker of dietary intake. Prog Lipid Res. 2008;47(5):348–380. 10.1016/J.PLIPRES.2008.03.003 18435934

[10] Farina EK, Kiel DP, Roubenoff R, Schaefer EJ, Cupples LA, Tucker KL. Plasma phosphatidylcholine concentrations of polyunsaturated fatty acids are differentially associated with hip bone mineral density and hip fracture in older adults: the Framingham osteoporosis study. J Bone Miner Res. 2012;27(5):1222–1230. 10.1002/JBMR.1581 22392875 PMC3565380

[11] Eriksson S, Mellstrm D, Strandvik B. Fatty acid pattern in serum is associated with bone mineralisation in healthy 8-year-old children. Br J Nutr. 2009;102(3):407–412. 10.1017/S0007114508190286 19175947

[12] Garemo M, Sundh V, Mellström D, Strandvik B. Serum phospholipid fatty acids are associated with bone mass in healthy 4-years-old children. Prostaglandins Leukot Essent Fatty Acids. 2024;200:102606. 10.1016/J.PLEFA.2023.102606 38181601

[13] Högström M, Nordström P, Nordström A. n-3 Fatty acids are positively associated with peak bone mineral density and bone accrual in healthy men: the NO2 study. Am J Clin Nutr. 2007;85(3):803–807. 10.1093/AJCN/85.3.803 17344503

[14] Damsgaard CT, Mølgaard C, Matthiessen J, Gyldenløve SN, Lauritzen L. The effects of n-3 long-chain polyunsaturated fatty acids on bone formation and growth factors in adolescent boys. Pediatr Res. 2012;71(6):713–719. 10.1038/PR.2012.28 22337227

[15] Lakka TA, Lintu N, Väistö J, et al. A 2 year physical activity and dietary intervention attenuates the increase in insulin resistance in a general population of children: the PANIC study. Diabetologia. 2020;63(11):2270–2281. 10.1007/s00125-020-05250-0 32816094 PMC7527318

[16] Sallinen T, Viitasalo A, Lintu N, et al. The effects of an 8-year individualised lifestyle intervention on food consumption and nutrient intake from childhood to adolescence: the PANIC Study. J Nutr Sci. 2022;11:e40. 10.1017/jns.2022.13 35720174 PMC9171599

[17] Kaitosaari T, Rönnemaa T, Viikari J, et al. Low-saturated fat dietary counseling starting in infancy improves insulin sensitivity in 9-year-old healthy children: the special Turku coronary risk factor intervention project for children (STRIP) study. Diabetes Care. 2006;29(4):781–785. 10.2337/DIACARE.29.04.06.DC05-1523 16567815

[18] Venäläinen TM, Viitasalo AM, Schwab US, et al. Effect of a 2-y dietary and physical activity intervention on plasma fatty acid composition and estimated desaturase and elongase activities in children: the physical activity and nutrition in children study. Am J Clin Nutr. 2016;104(4):964–972. 10.3945/AJCN.116.136580 27581473

[19] Agren JJ, Julkunen A, Penttila I. Rapid separation of serum lipids for fatty acid analysis by a single aminopropyl column. J Lipid Res. 1992;33(12):1871–1876. 10.1016/S0022-2275(20)41345-8 1479296

[20] Vessby B, Gustafsson IB, Tengblad S, Berglund L. Indices of fatty acid desaturase activity in healthy human subjects: effects of different types of dietary fat. Br J Nutr. 2013;110(5):871–879. 10.1017/S0007114512005934 23414551

[21] Warensjö E, Risérus U, Gustafsson IB, Mohsen R, Cederholm T, Vessby B. Effects of saturated and unsaturated fatty acids on estimated desaturase activities during a controlled dietary intervention. Nutr Metab Cardiovasc Dis. 2008;18(10):683–690. 10.1016/J.NUMECD.2007.11.002 18367385

[22] Mahendran Y, Ågren J, Uusitupa M, et al. Association of erythrocyte membrane fatty acids with changes in glycemia and risk of type 2 diabetes. Am J Clin Nutr. 2014;99(1):79–85. 10.3945/AJCN.113.069740 24153340

[23] Saari A, Sankilampi U, Hannila ML, Kiviniemi V, Kesseli K, Dunkel L. New Finnish growth references for children and adolescents aged 0 to 20 years: length/height-for-age, weight-for-length/height, and body mass index-for-age. Ann Med. 2011;43(3):235–248. 10.3109/07853890.2010.515603 20854213

[24] Marshall WA, Tanner JM. Variations in pattern of pubertal changes in girls. Arch Dis Child. 1969;44(235):291–303. 10.1136/ADC.44.235.291 5785179 PMC2020314

[25] Marshall WA, Tanner JM. Variations in the pattern of pubertal changes in boys. Arch Dis Child. 1970;45(239):13–23. 10.1136/ADC.45.239.13 5440182 PMC2020414

[26] Moore SA, McKay HA, Macdonald H, et al. Enhancing a somatic maturity prediction model. Med Sci Sports Exerc. 2015;47(8):1755–1764. 10.1249/MSS.0000000000000588 25423445

[27] Soininen S, Eloranta AM, Schwab U, Lakka TA. Sources of vitamin D and determinants of serum 25-hydroxyvitamin D in Finnish adolescents. Eur J Nutr. 2023;62(2):1011–1025. 10.1007/S00394-022-03039-Y 36350359 PMC9941269

[28] Soininen S, Eloranta AM, Lindi V, et al. Determinants of serum 25-hydroxyvitamin D concentration in Finnish children: the physical activity and nutrition in children (PANIC) study. Br J Nutr. 2016;25(6):1–12. 10.1017/S0007114515005292 26836317

[29] Soininen S, Sidoroff V, Lindi V, et al. Body fat mass, lean body mass and associated biomarkers as determinants of bone mineral density in children 6–8 years of age – the physical activity and nutrition in children (PANIC) study. Bone. 2018;108:106–114. 10.1016/j.bone.2018.01.003 29307776

[30] Eloranta AMM, Venäläinen T, Soininen S, et al. Food sources of energy and nutrients in Finnish girls and boys 6-8 years of age - the PANIC study. Food Nutr Res. 2016;60(1):32444. 10.3402/fnr.v60.32444 27702428 PMC5045967

[31] Watkins BA, Lippman HE, Le Bouteiller L, Li Y, Seifert MF. Bioactive fatty acids: role in bone biology and bone cell function. Prog Lipid Res. 2001;40(1-2):125–148. 10.1016/S0163-7827(00)00016-3 11137570

[32] Roncero-Martín R, Aliaga I, Moran JM, et al. Plasma fatty acids and quantitative ultrasound, DXA and pQCT derived parameters in postmenopausal Spanish women. Nutrients. 2021;13(5):154. 10.3390/NU13051454 PMC8146540 33922947

[33] Paunescu AC, Ayotte P, Dewailly E, Dodin S. Saturated and monounsaturated fatty acid status is associated with bone strength estimated by calcaneal ultrasonography in Inuit women from Nunavik (Canada): a cross-sectional study. J Nutr Health Aging. 2014;18(7):663–671. 10.1007/S12603-014-0498-025226104

[34] Venäläinen T, Ågren J, Schwab U, et al. Cross-sectional associations of plasma fatty acid composition and estimated desaturase and elongase activities with cardiometabolic risk in Finnish children--the PANIC study. J Clin Lipidol. 2016;10(1):82–91. 10.1016/J.JACL.2015.09.004 26892124

[35] Weitkunat K, Schumann S, Nicke D, et al. Odd-chain fatty acids as a biomarker for dietary fiber intake: a novel pathway for endogenous production from propionate. Am J Clin Nutr. 2017;105(6):1544–1551. 10.3945/AJCN.117.152702 28424190

[36] Venäläinen T, Schwab U, Ågren J, et al. Cross-sectional associations of food consumption with plasma fatty acid composition and estimated desaturase activities in Finnish children. Lipids. 2014;49(5):467–479. 10.1007/S11745-014-3894-7 24659110

[37] Yuan S, Lemming EW, Michaëlsson K, Larsson SC. Plasma phospholipid fatty acids, bone mineral density and fracture risk: evidence from a Mendelian randomization study. Clin Nutr. 2020;39(7):2180–2186. 10.1016/J.CLNU.2019.09.005 31564377

[38] Kruger MC, Coetzee M, Haag M, Weiler H. Long-chain polyunsaturated fatty acids: selected mechanisms of action on bone. Prog Lipid Res. 2010;49(4):438–449. 10.1016/J.PLIPRES.2010.06.002 20600307

[39] Bellissimo MP, Ziegler TR, Jones DP, et al. Plasma high-resolution metabolomics identifies linoleic acid and linked metabolic pathways associated with bone mineral density. Clin Nutr. 2021;40(2):467–475. 10.1016/J.CLNU.2020.05.041 32620447 PMC7714706

[40] Liang H, Xiong C, Luo Y, et al. Association between serum polyunsaturated fatty acids and bone mineral density in US adults: NHANES 2011-2014. Front Endocrinol (Lausanne). 2023;14:1266329. 10.3389/FENDO.2023.1266329 38047106 PMC10690584

[41] Vidgren HM, Ågren JJ, Schwab U, Rissanen T, Hänninen O, Uusitupa MIJ. Incorporation of n-3 fatty acids into plasma lipid fractions, and erythrocyte membranes and platelets during dietary supplementation with fish, fish oil, and docosahexaenoic acid-rich oil among healthy young men. Lipids. 1997;32(7):697–705. 10.1007/S11745-997-0089-X 9252957

[42] Mercola J, D’Adamo CR. Linoleic acid: a narrative review of the effects of increased intake in the standard American diet and associations with chronic disease. Nutrients. 2023;15(14):3129. 10.3390/NU15143129 37513547 PMC10386285

[43] Tao J, Shi J, Lu Y, et al. Overexpression of stearoyl-CoA desaturase 1 in bone-marrow mesenchymal stem cells increases osteogenesis. Panminerva Med. 2013;55(3):283–289. 24088802

[44] Rios-Esteves J, Resh MD. Stearoyl CoA desaturase is required to produce active, lipid-modified Wnt proteins. Cell Rep. 2013;4(6):1072–1081. 10.1016/J.CELREP.2013.08.027 24055053 PMC3845236

[45] Kragl A, Hannemann A, Nauck M, et al. Genetic variants in WNT16 and PKD2L1 locus affect heel ultrasound bone stiffness: analyses from the general population and patients evaluated for osteoporosis. Calcif Tissue Int. 2023;113(5):540–551. 10.1007/S00223-023-01141-9 37831088 PMC10618371

[46] Melhus H, Risérus U, Warensjö E, et al. A high activity index of stearoyl-CoA desaturase is associated with increased risk of fracture in men. Osteoporos Int. 2008;19(7):929–934. 10.1007/S00198-007-0521-Y 18066610 PMC2440922

[47] The International Society for Clinical Densitometry . 2019 ISCD Official Positions Pediatric. Accessed March 9, 2025. https://iscd.org/wp-content/uploads/2021/09/2019-Official-Positions-Pediatric-1.pdf

[48] Crabtree NJ, Arabi A, Bachrach LK, et al. Dual-energy X-ray absorptiometry interpretation and reporting in children and adolescents: the revised 2013 ISCD pediatric official positions. J Clin Densitom. 2014;17(2):225–242. 10.1016/j.jocd.2014.01.003 24690232

[49] Margulies L, Horlick M, Thornton JC, Wang J, Ioannidou E, Heymsfield SB. Reproducibility of pediatric whole body bone and body composition measures by dual-energy X-ray absorptiometry using the GE lunar prodigy. J ClinDensitom. 2005;8(1094-6950 (Print)):298–304. 10.1385/jcd:8:3:298 16055960

