# Supplementary material for: Plasma fatty acid composition predicts bone mineral accrual from childhood to adolescence: the Physical Activity and Nutrition in Children study
Source: J Bone Miner Res. 2025 Aug 1;40(11):1265–77. doi: 10.1093/jbmr/zjaf104 (PMC12578281; doi:10.1093/jbmr/zjaf104)
Supplement: Lakka_et_al_Supplemental_Table_revised_clean_zjaf104 [file lakka_et_al_supplemental_table_revised_clean_zjaf104.docx]

**Supplemental Table. Longitudinal associations of fatty acids in plasma phospholipids with bone mineral density over 8 years after adjustments for dietary calcium and vitamin D intake in 441 children with complete dietary intake data.**

|  | **Model 1** | | **Model 2** | | **Model 3** | |
| --- | --- | --- | --- | --- | --- | --- |
| **Fatty acids (mol%)** | **β** | **p-value** | **β** | **p-value** | **β** | **p-value** |
| **Total SFA** | 0.013 | 0.069 | 0.011 | 0.130 | 0.013 | 0.070 |
| 14:0, Myristic acid | 0.002 | 0.707 | 0.000 | 0.986 | 0.003 | 0.628 |
| 15:0, Pentadecanoic acid | 0.003 | 0.578 | 0.001 | 0.920 | 0.004 | 0.489 |
| 16:0, Palmitic acid | -0.002 | 0.844 | -0.002 | 0.842 | 0.000 | 0.983 |
| 17:0, Margaric acid | 0.010 | 0.220 | 0.006 | 0.462 | 0.012 | 0.147 |
| 18:0, Stearic acid | 0.013 | 0.088 | 0.012 | 0.137 | 0.010 | 0.188 |
| 20:0, Arachidic acid | 0.018 | **0.029** | 0.017 | **0.037** | 0.020 | **0.018** |
| 22:0, Behenic acid | 0.012 | 0.135 | 0.012 | 0.143 | 0.013 | 0.090 |
| 24:0, Lignoceric acid | 0.008 | 0.296 | 0.008 | 0.316 | 0.010 | 0.213 |
| **Total MUFA** | -0.009 | 0.230 | -0.007 | 0.311 | -0.008 | 0.256 |
| 16:1n-7, Palmitoleic acid | 0.013 | 0.069 | 0.013 | 0.067 | 0.014 | 0.052 |
| 18:1n-9, Oleic acid | -0.012 | 0.088 | -0.010 | 0.132 | -0.012 | 0.079 |
| 18:1n-7, Cis-vaccenic acid | -0.007 | 0.412 | -0.007 | 0.394 | -0.004 | 0.593 |
| 20:1n-9 +11, Eicosenoic acid | -0.016 | **0.029** | -0.016 | **0.025** | -0.015 | **0.040** |
| 24:1n-9, Nervonic acid | 0.016 | **0.035** | 0.016 | **0.039** | 0.019 | **0.017** |
| **Total PUFA** | -0.001 | 0.879 | -0.001 | 0.907 | -0.001 | 0.842 |
| *Total n-6 PUFA* | -0.007 | 0.288 | -0.007 | 0.294 | -0.007 | 0.285 |
| 18:2n-6, Linoleic acid | -0.019 | **0.012** | -0.019 | **0.011** | -0.020 | **0.008** |
| 20:3n-6, Dihomo-gamma-linolenic acid | 0.026 | **0.002** | 0.025 | **0.003** | 0.027 | **0.001** |
| 20:4n-6, Arachidonic acid | 0.014 | 0.094 | 0.015 | 0.073 | 0.015 | 0.070 |
| 22:4n-6, Adrenic acid | 0.008 | 0.354 | 0.009 | 0.276 | 0.011 | 0.199 |
| 22:5n-6, Osbond acid | -0.012 | 0.216 | -0.010 | 0.290 | -0.008 | 0.398 |
| *Total n-3 PUFA* | 0.009 | 0.211 | 0.009 | 0.202 | 0.009 | 0.230 |
| 18:3n-3, Alpha-linolenic acid | -0.002 | 0.801 | -0.002 | 0.742 | -0.002 | 0.758 |
| 20:5n-3, Eicosapentaenoic acid, EPA | 0.006 | 0.367 | 0.005 | 0.441 | 0.005 | 0.464 |
| 22:5n-3, Docosapentaenoic acid, DPA | 0.020 | **0.023** | 0.019 | **0.024** | 0.022 | **0.011** |
| 22:6n-3, Docosahexaenoic acid, DHA | 0.007 | 0.374 | 0.008 | 0.309 | 0.007 | 0.382 |
| *n-6/n-3 PUFA ratio* | -0.013 | 0.091 | -0.013 | 0.097 | -0.012 | 0.119 |
| **Desaturase and elongase activities** |  |  |  |  |  |  |
| Stearoyl-CoA-desaturase, SCD | 0.014 | 0.052 | 0.014 | 0.050 | 0.015 | **0.040** |
| Δ5-desaturase, D5D | -0.010 | 0.217 | -0.008 | 0.287 | -0.009 | 0.223 |
| Δ6-desaturase, D6D | 0.029 | **<0.001** | 0.028 | **<0.001** | 0.030 | **<0.001** |
| Elongase | -0.017 | **0.042** | -0.016 | **0.043** | -0.017 | **0.039** |

The values are standardized regression coefficients from linear mixed effects models adjusted for sex, maturity offset, and follow-up time (Model 1); sex, maturity offset, follow-up time, and dietary calcium intake (Model 2); sex, maturity offset, follow-up time, and dietary vitamin D intake (Model 3). Statistically significant values are bolded.
